# Cardiovascular and Metabolic Disease: New Treatments and Future Directions 2.0

**DOI:** 10.3390/biomedicines12061356

**Published:** 2024-06-18

**Authors:** Alfredo Caturano

**Affiliations:** 1 Department of Advanced Medical and Surgical Sciences, University of Campania Luigi Vanvitelli, I-80138 Naples, Italy; alfredo.caturano@unicampania.it; Tel.: +39-3338616985; 2 Department of Experimental Medicine, University of Campania Luigi Vanvitelli, I-80138 Naples, Italy

## 1. Introduction

Over recent decades, cardiovascular diseases (CVDs) and metabolic disorders have emerged as major global health challenges, exacting a heavy toll on human lives and burdening healthcare systems worldwide. Despite advancements in medical science and technology, these conditions persist as leading causes of morbidity and mortality, prompting a critical need for innovative approaches to prevention, diagnosis, and treatment [1,2].

The intersection of cardiovascular diseases and metabolic disorders is complex and multifaceted. The epidemiological landscape reveals a stark reality: a significant proportion of CVD-related deaths are intricately linked to the coexistence of metabolic ailments, particularly diabetes. This nexus demands comprehensive understanding and targeted interventions to mitigate the intertwined risks and complexities inherent in these conditions [3,4].

In the year 2019 alone, an alarming 17.9 million lives were lost to cardiovascular diseases, constituting a staggering 32% of all global deaths [5]. Heart attacks and strokes accounted for a substantial portion of these fatalities, underscoring the urgent need for effective preventive strategies and therapeutic interventions. Moreover, the burden of metabolic diseases, exemplified by the pervasive prevalence of diabetes, adds to the gravity of the situation. In 2019, global diabetes prevalence stood at 9.3%, affecting approximately 463 million individuals worldwide. Projections indicate a steady rise in prevalence, with estimates indicating a rise to 10.2% (578 million) by 2030 and 10.9% (700 million) by 2045 [6]. Alarmingly, 1.5 million deaths are directly attributed to diabetes annually, accentuating the imperative for the early detection and comprehensive management of these interrelated conditions [7].

Behavioral risk factors such as tobacco use, unhealthy dietary patterns, sedentary lifestyles, and excessive alcohol consumption exacerbate the susceptibility to both cardiovascular diseases and metabolic disorders. Therefore, addressing these modifiable risk factors assumes paramount importance in the overarching strategy for disease prevention and control [8,9,10].

In the realm of therapeutics, recent years have witnessed significant strides in the management of cardiovascular and metabolic diseases. From pharmacological innovations, including the advent of sodium glucose cotransporter 2 inhibitors heralding promising outcomes in heart failure and diabetes management, to advancements in interventional techniques such as immediate revascularization for acute myocardial infarction and cerebral infarction, the landscape of cardiovascular care continues to evolve rapidly [11,12]. Additionally, emerging modalities like mechanical cardiac support and multiorgan transplantation offer novel avenues for managing the most complex manifestations of heart failure, underscoring the transformative potential of cutting-edge interventions [11,13].

This Special Issue has captured the diversity of the studies that focus on the latest scientific insights and technological innovations in the realm of cardiovascular and metabolic disease management.

## 2. Overview of Published Articles

These articles provide a diverse range of insights into cardiovascular and metabolic diseases, reflecting the multifaceted nature of these conditions and the ongoing efforts to better understand and treat them.

Seung Eun Jung et al. (contribution 1) investigated the role of exosomal RNAs, particularly microRNAs, in animal models of acute myocardial infarction (AMI). By identifying differentially expressed miRNAs and validating their functions in vitro, the research enhances our understanding of post-AMI molecular changes and explores the potential of exoRNAs as biomarkers or therapeutic targets.

Federica Fogacci et al. (contribution 2) focused on the effects of Evolocumab, a PCSK9 inhibitor. In this study, the researchers examined how lipoprotein(a) and low-density lipoprotein cholesterol respond to treatment over time, particularly considering sex-related differences. The understanding of these dynamics could contribute to personalized treatment strategies for cardiovascular disease.

Alfredo Caturano et al. (contribution 3) investigated the association between the fatty liver index (FLI) and cardiovascular events in liver transplant recipients; this study underscores the importance of monitoring metabolic parameters and liver health in this unique cohort of patients for cardiovascular disease prevention, suggesting FLI as a potential predictive marker.

Irina Tarasova et al. (contribution 4) evaluated different cognitive training approaches in cardiac surgery patients, highlighting the potential benefits of multi-tasking training for cognitive rehabilitation in the postoperative period.

Teodor Salmen et al. (contribution 5) assessed the efficacy of antidiabetic medications, particularly SGLT-2 inhibitors and GLP-1 receptor agonists, in real-life clinical practice. This study provides valuable insights into their effectiveness in managing type 2 diabetes mellitus, especially when used in conjunction with other medications.

Juan Carlos Sánchez-Delgado et al. (contribution 6) investigated the association between handgrip strength and vascular function in individuals with metabolic syndrome. This study highlights the potential role of muscle strength in mitigating vascular dysfunction, particularly in older adults.

Maria Kercheva et al. (contribution 7) found that kidneys of patients with fatal myocardial infarction (MI) showed a predominance of CD163+ macrophages, while controls without cardiovascular diseases (CVD) had a higher presence of CD163+, CD206+, and CD68+ macrophages. In MI patients, CD80+ and CD206+ macrophages exhibited a biphasic response, decreasing over time post-MI.

Anna Kurpas et al. (contribution 8) found that epicardial global circumferential strain measured using 2D speckle-tracking echocardiography significantly correlated with serum FGF23 levels in patients with type 2 diabetes mellitus, suggesting FGF23 as a potential early marker of myocardial damage in these patients. Additionally, patients with left ventricular diastolic dysfunction had lower estimated glomerular filtration rates and higher hemoglobin A1c levels.

Natalia Beloborodova et al. (contribution 9) found that higher levels of sepsis-associated aromatic microbial metabolites in the blood before and shortly after surgery were linked to postoperative complications in patients with aortic aneurysm. This suggests that an impaired microbiota metabolism plays a significant role in postoperative outcomes, indicating a potential target for new prevention strategies.

Ioan Alexandru Balmos et al. (contribution 10) found that inflammation, microcalcification, and high-grade osteopontin expression are significantly associated with plaque ulceration and atherothrombosis in patients with carotid artery stenosis, indicating their critical roles in plaque formation and destabilization. Higher osteopontin expression was also linked to the presence of a lipid core, suggesting its importance in plaque progression.

Moustapha Agossou et al. (contribution 11) showed the results of a brief report assessing the impact of previous continuous positive airway pressure (CPAP) use on the quality of noninvasive ventilation (NIV) in patients with obesity hypoventilation syndrome. They found no significant difference in NIV quality between patients with and without prior CPAP use.

Preeti Kumari Chaudhary et al. (contribution 12) reviewed the literature exploring how platelet proteomics can advance the diagnosis and treatment of cardiovascular thromboembolic diseases and cancer. By analyzing peptides and proteins in platelets, researchers can identify disease-specific biomarkers for personalized medicine.

Esther Solano-Pérez et al. (contribution 13) discuss how obstructive sleep apnea (OSA) in children can increase cardiovascular risk. Their review proposes using echocardiography alongside polysomnography to assess cardiac function and structure in OSA patients for better risk management.

Silvia Preda et al. (contribution 14) presented a case study of transcatheter aortic valve implantation (TAVI) in a patient who had previously undergone a heart transplant. It highlights the challenges and potential benefits of using TAVI in heart transplant recipients.

Giovanni Cimmino et al. (contribution 15) focused on analyzing literature data with the aim of exploring emerging non-traditional risk factors for cardiovascular disease and their potential impact on disease development. They discuss how these factors may influence current cardiovascular risk assessment models.

Weronika Frąk et al. (contribution 16) discuss the therapeutic potential of sodium/glucose cotransporter 2 inhibitors in treating cardiovascular diseases. They report the available data on the efficacy of these medications in improving cardiovascular outcomes.

Ayodeji A. Olabiyi et al. (contribution 17) focus their review article on examining the use of dietary interventions and medicinal herbs in treating cardiovascular disease. They propose combining these approaches with pharmaceutical drugs for more effective treatment strategies.

Simonetta Genovesi et al. (contribution 18) dealt with lipoprotein(a) (Lp(a)) as a marker of cardiovascular health risk in young populations. They explore the evidence surrounding Lp(a) and its potential role in assessing cardiovascular risk in children and adolescents.

Ozan Demirel et al. (contribution 19) examined different serum biomarkers for predicting atrial fibrillation recurrence after electrical cardioversion in their review article. They discussed the potential of these biomarkers in improving patient outcomes.

Polyxeni Mantzouratou et al. (contribution 20) performed a literature review on the role of thyroid hormone signaling in cardiac repair and regeneration, reporting that thyroid hormone therapy may benefit heart failure patients.

## 3. Future Directions

Looking ahead, the landscape of cardiovascular and metabolic disease research holds immense promise for transformative advancements and innovative interventions. As we navigate the complexities of these interconnected conditions, several key avenues emerge as focal points for future exploration and inquiry.

First and foremost, the imperative for personalized medicine in cardiovascular and metabolic disease management is looming large on the horizon [14]. With the advent of precision medicine and genomic technologies, there exists unprecedented potential to tailor interventions to the individual characteristics and needs of patients, thereby optimizing therapeutic outcomes and minimizing adverse effects. Harnessing the power of big data analytics and artificial intelligence, researchers can glean valuable insights into the intricate interplay of genetic, environmental, and lifestyle factors influencing disease susceptibility and progression, paving the way for more targeted and effective treatment strategies [15].

Furthermore, the pursuit of novel therapeutic targets and modalities represents a frontier ripe for exploration. From elucidating the molecular mechanisms underlying disease pathogenesis to identifying druggable targets and developing innovative therapeutics, ongoing research efforts hold promise for revolutionizing the treatment landscape for cardiovascular and metabolic disorders. Emerging areas such as gene editing, stem cell therapy, and regenerative medicine offer tantalizing prospects for disease modification and regeneration, offering new hope for patients with refractory conditions and advanced disease states [16,17].

In parallel, efforts to address the social determinants of health and reduce health disparities remain paramount [18]. Recognizing the profound impact of socioeconomic factors, access to care, and healthcare disparities on disease outcomes, future initiatives must prioritize equitable access to preventive services, early detection, and evidence-based interventions for all segments of the population. By addressing structural barriers and fostering community engagement, healthcare stakeholders can empower individuals to make informed choices and adopt healthy behaviors, thereby mitigating the burden of cardiovascular and metabolic diseases on vulnerable populations. Moreover, the integration of digital health technologies and telemedicine holds immense potential for enhancing disease management and improving patient outcomes. From remote monitoring and teleconsultation to wearable devices and mobile health applications, digital innovations offer unprecedented opportunities to empower patients, enhance care coordination, and facilitate real-time data-driven decision-making. By harnessing the power of technology to bridge geographical barriers, streamline healthcare delivery, and empower patients to actively participate in their own care, we can unlock new frontiers in the prevention and management of cardiovascular and metabolic diseases [19].

In conclusion, the future of cardiovascular and metabolic disease research is characterized by boundless possibilities and transformative potential. By embracing a multidisciplinary approach, leveraging cutting-edge technologies, and prioritizing patient-centered care, we can chart a course towards a future where the burden of these devastating conditions is alleviated, and the promise of improved health and well-being becomes a tangible reality for individuals and communities worldwide.

## Data Availability

No dataset was generated for the publication of this article.
